# Improved adherence to statin treatment and differences in results between men and women after pictorial risk communication—a sub-study of the VIPVIZA RCT

**DOI:** 10.1007/s00228-024-03694-6

**Published:** 2024-04-29

**Authors:** Henrik Holmberg, Eva-Lotta Glader, Ulf Näslund, Bo Carlberg, Eva Sönnerstam, Margareta Norberg, Anders Själander

**Affiliations:** 1 Department of Public Health and Clinical Medicine, Umeå University, 907 36 Umeå, Sweden; 2 Department of Epidemiology and Global Health, Umeå University, 907 36 Umeå, Sweden

**Keywords:** Statin initiation, Statins, Cardiovascular risk, Atherosclerosis, Cardiovascular disease prevention, Pictorial information

## Abstract

**Background:**

People with intermediate CVD risk constitute most of the population. Within this group, the proportion of events is lower compared to the high-risk group, but they contribute with the largest absolute number of events. Atherosclerosis is a dynamic process and progression can be slowed or even reversed with medication and lifestyle changes, but adherence to prescribed treatment is crucial.

**Aim:**

To investigate the long-term effects of interventions with pictorial risk communication of cardiovascular (CVD) risk on average adherence in a group of statin users. Compare response in adherence over time between men and women after intervention.

**Methods:**

Participants on active statin treatment were followed up to 5 years after being randomly assigned to an intervention program aimed at raising CVD risk awareness among participants and their physicians. Merging prescribed medication databases with VIPVIZA study to study adherence over time. A moving average adherence was used to compare groups.

**Results:**

Generally, the average adherence to statins among the 512 participants was high. Men had a higher average adherence over time, while women had a sharper increase in adherence in conjuncture with the intervention program.

**Conclusions:**

Both men and women were receptive to pictorial information regarding CVD risk, but the intervention effect was more pronounced in women. Sex differences are important when considering risk communication strategies. Periodically repeating the intervention was beneficial for maintaining the intervention effect over time.

**Trial registration:**

The VIPVIZA study is registered with ClinicalTrials.gov, May 8, 2013, number NCT01849575.

## Introduction

Non-adherence is estimated to cost the healthcare system $100–300 billion every year in the USA alone; globally, the numbers are even greater [1]. The WHO’s definition of adherence is “Adherence is the extent to which a person’s behaviour – taking medication, following a diet, and/or executing lifestyle changes – corresponds with agreed recommendations from a health care provider” [2]*.* Statin prescriptions to reduce CVD risk are common in both primary and secondary prevention, and the efficacy of statins in reducing CVD mortality is widely accepted [3].

The effectiveness of a medical treatment is heavily dependent on the patient’s adherence. Compared with men, women are on average less likely to adhere to statin treatment, both in primary and secondary prevention [3, 4]. Part of that difference is due to that women and the treating physician perceive the women to be at lower risk, compared to men with similar characteristics [5]. There is a need for improved CVD-prevention implementation in clinical practice [6] and for physicians to prompt and personalise the intervention, target patients at risk of non-adherence, and improve motivation and communication [7].

VIPVIZA is a randomised controlled intervention study targeting both participants and their respective physician. The VIPVIZA interventions is personalised and includes colour-coded and age-related pictorial risk information based on the individual’s ultrasound examination of the carotid arteries and a follow-up motivational dialogue with a specially trained nurse. It has been previously studied how the intervention affect physicians and participants behaviours and outcomes and time to initiation of statins [8–11]. The most recently published results from VIPVIZA, with an example of the pictorial risk communication, timeline for the intervention and as supplement, a translation to English of the written information in the ultrasound report [12].

## Objectives

To evaluate adherence to statins over 5 years in VIPVIZA and investigate the long-term effect of the VIPVIZA interventions on average adherence in statin users and to compare differences in adherence between men and women over time after the intervention.

## Method

### VIP, recruitment base

In the Västerbotten intervention programme (VIP), all inhabitants in the county of Västerbotten aged 40, 50, or 60 years were invited to a health examination with CVD risk factor screening followed by a motivational interview aimed to promote lifestyle adjustments and pharmacological preventive treatment according to clinical guidelines [13]. Participation rates during the inclusion period April 2013–May 2016 were 59–69%, corresponding to 6500–7000 participants yearly. Only a small social selection bias has been previously reported [14].

### Participants VIPVIZA

Participants, who fulfilled the inclusion criteria, were invited to join the VIPVIZA trial after the VIP motivational interview. The inclusion criteria for VIPVIZA were as follows:

1. 60 years of age
2. 50 years of age with at least one of the following risk factors: diabetes, smoking, hypertension, LDL-cholesterol > 4.5 mmol/L, abdominal obesity or first degree relative with CVD history before 60 years of age
3. 40 years old with first-degree relative with CVD history before 60 years of age

Of the VIP-population, 61% were eligible for VIPVIZA, and in this study, only 50 and 60 years old were included.

In the VIPVIZA baseline assessment, asymptomatic atherosclerotic disease was identified by carotid ultrasound examination, measuring carotid intima media thickness (cIMT) as well as the presence of carotid plaque. Before the ultrasound examination, participants were randomised 1:1 to the intervention or control group, but the randomization was concealed to participants and the ultrasound operator. Details about the VIPVIZA procedures have been previously published [8, 10, 12].

### Study population

**Fig. 1:**
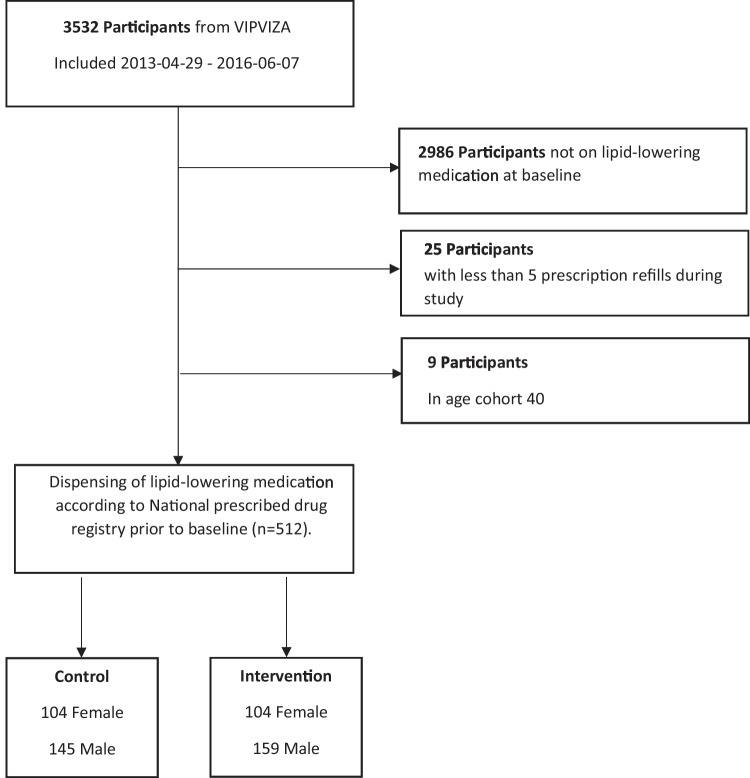
Flowchart illustrating exclusion and group eligible for analysis

Of the 4177 invited individuals, 3532 were enrolled in VIPVIZA. Five hundred forty-six of those met the criteria “active statin treatment during the last 270 days before baseline examination”. Thirty-four participants were excluded; see Table 1 for participant characteristics and Fig. 1 for flow chart. The VIPVIZA participants in the present study, those on statins prior to baseline, generally had higher cholesterol levels and more risk factors than the excluded participants. As all 512 participants in the final analysis were on statin treatment, adherence analyses could be performed without time lag to initiation.

**Table 1:** Background characteristics of study population by sex and group

	**Male**		**Female**		**Overall**	
	**Control**	**Intervention**	**Control**	**Intervention**	**Control**	**Intervention**
	**(*N* = 145)**	**(*N* = 159)**	**(*N* = 104)**	**(*N* = 104)**	**(*N* = 249)**	**(*N* = 263)**
**Age (group)**						
50	26 (17.9%)	32 (20.1%)	10 (9.6%)	10 (9.6%)	36 (14.5%)	42 (16.0%)
60	119 (82.1%)	127 (79.9%)	94 (90.4%)	94 (90.4%)	213 (85.5%)	221 (84.0%)
**BMI**						
Mean (SD)	29.5 (4.36)	29.1 (4.13)	29.5 (5.78)	27.9 (4.86)	29.5 (4.99)	28.6 (4.46)
Median [Min, Max]	28.7 [21.2, 43.1]	28.7 [22.1, 48.4]	28.6 [17.7, 49.9]	26.5 [20.3, 39.3]	28.7 [17.7, 49.9]	27.9 [20.3, 48.4]
**Weight (kg)**						
Mean (SD)	93.0 (14.7)	92.0 (16.4)	78.2 (16.2)	74.4 (13.6)	86.8 (17.0)	85.0 (17.6)
Median [Min, Max]	92.0 [62.0, 139]	90.0 [61.0, 172]	75.0 [50.0, 131]	71.5 [54.0, 117]	87.0 [50.0, 139]	83.0 [54.0, 172]
**Waist (cm)**						
Mean (SD)	105 (10.3)	104 (11.4)	99.3 (14.0)	94.3 (11.8)	102 (12.2)	100 (12.6)
Median [Min, Max]	104 [80.0, 137]	102 [84.0, 149]	98.0 [65.0, 142]	93.0 [69.0, 129]	101 [65.0, 142]	99.0 [69.0, 149]
**Systolic BP (mm Hg)**						
Mean (SD)	132 (14.6)	133 (18.0)	130 (15.0)	129 (15.5)	131 (14.7)	132 (17.2)
Median [min, max]	130 [100, 185]	132 [95.0, 230]	130 [97.0, 172]	128 [97.0, 180]	130 [97.0, 185]	130 [95.0, 230]
**Diastolic BP (mm Hg)**						
Mean (SD)	83.0 (9.53)	85.1 (12.0)	81.2 (8.38)	81.8 (8.67)	82.2 (9.10)	83.8 (10.9)
Median [min, max]	84.0 [59.0, 112]	85.0 [60.0, 140]	80.0 [54.0, 102]	80.0 [60.0, 110]	82.0 [54.0, 112]	83.0 [60.0, 140]
**LDL (mmol/L)**						
Mean (SD)	2.77 (1.13)	2.84 (1.11)	3.06 (1.26)	3.24 (1.27)	2.89 (1.19)	3.00 (1.19)
Median [min, max]	2.60 [0.800, 7.30]	2.60 [0.90, 6.20]	2.70 [1.30, 6.70]	3.00 [1.40, 6.90]	2.60 [0.800, 7.30]	2.80 [0.90, 6.90]
Missing	9 (6.2%)	8 (5.0%)	3 (2.9%)	2 (1.9%)	12 (4.8%)	10 (3.8%)
**HDL (mmol/L)**						
Mean (SD)	1.16 (0.306)	1.20 (0.333)	1.43 (0.428)	1.47 (0.544)	1.27 (0.385)	1.30 (0.448)
Median [min, max]	1.11 [0.600, 2.20]	1.13 [0.72, 2.50]	1.39 [0.730, 3.26]	1.40 [0.60, 5.00]	1.23 [0.60, 3.26]	1.20 [0.60, 5.00]
**Education**						
Basic to mid-level	111 (76.6%)	122 (76.7%)	66 (63.5%)	67 (64.4%)	177 (71.1%)	189 (71.9%)
High	33 (22.8%)	35 (22.0%)	37 (35.6%)	36 (34.6%)	70 (28.1%)	71 (27.0%)
**SCORE (risk)**						
Low (< 1%)	23 (15.9%)	25 (15.7%)	80 (76.9%)	84 (80.8%)	103 (41.4%)	109 (41.4%)
Moderate (1–4%)	118 (81.4%)	128 (80.5%)	24 (23.1%)	20 (19.2%)	142 (57.0%)	148 (56.3%)
High (5–9%)	3 (2.1%)	6 (3.8%)	0 (0%)	0 (0%)	3 (1.2%)	6 (2.3%)
Very high (≥ 10%)	0 (0%)	0 (0%)	0 (0%)	0 (0%)	0 (0%)	0 (0%)
**Framingham (risk)**						
Low (< 5%)	0 (0%)	1 (0.6%)	13 (12.5%)	9 (8.7%)	13 (5.2%)	10 (3.8%)
Light (5–9%)	12 (8.3%)	19 (11.9%)	33 (31.7%)	57 (54.8%)	45 (18.1%)	76 (28.9%)
Moderate (10–19%)	72 (49.7%)	68 (42.8%)	44 (42.3%)	28 (26.9%)	116 (46.6%)	96 (36.5%)
High (20–39%)	48 (33.1%)	60 (37.7%)	14 (13.5%)	10 (9.6%)	62 (24.9%)	70 (26.6%)
Very high (≥ 40%)	12 (8.3%)	11 (6.9%)	0 (0%)	0 (0%)	12 (4.8%)	11 (4.2%)
**VIPVIZA (vascular age)**						
Green	19 (13.1%)	17 (10.7%)	5 (4.8%)	11 (10.6%)	24 (9.6%)	28 (10.6%)
Yellow	28 (19.3%)	28 (17.6%)	20 (19.2%)	13 (12.5%)	48 (19.3%)	41 (15.6%)
Orange	38 (26.2%)	42 (26.4%)	26 (25.0%)	39 (37.5%)	64 (25.7%)	81 (30.8%)
Red	60 (41.4%)	72 (45.3%)	53 (51.0%)	41 (39.4%)	113 (45.4%)	113 (43.0%)
**Plaque detected**						
No	44 (30.3%)	58 (36.5%)	45 (43.3%)	40 (38.5%)	89 (35.7%)	98 (37.3%)
Yes	101 (69.7%)	101 (63.5%)	59 (56.7%)	64 (61.5%)	160 (64.3%)	165 (62.7%)

### Intervention

The results from the ultrasound baseline examination were used to compile the VIPVIZA pictorial risk information consisting of a graphical representation of atherosclerosis. A red circle represented presence and a green circle represented non-presence of plaque. A coloured gauge represented the vascular age based on measurement of carotid intima-media thickness versus actual (chronological) age. Vascular age was estimated in relation to subjects with the same sex and age in a reference population [8]. The gauge ranged from green to yellow, orange, and red, where the green sector corresponded to a vascular age at least − 10 years compared to chronological age and red corresponded to at least +10 years.

The intervention procedure consisted of three parts:

1. Mailing the result of the carotid ultrasound as a pictorial presentation within 2 weeks after examination, to participants and their respective physician in primary care. Written information was included to participants about the dynamic nature of atherosclerosis and opportunities to modify the progress through lifestyle change and pharmacological treatment [15 Supplementary material].
2. A follow-up phone call to the participant, in the intervention group, was made 2–4 weeks later by a research nurse, to reassure or answer questions as needed as well as a dialogue concerning measures for CVD prevention. To the family physician, information about the current guideline-based clinical significance of carotid ultrasound results was enclosed to all reports.
3. The pictorial information was repeated to the participant after 6 months.

The control group and their family physician did not receive this intervention at baseline. Details on the cognitive and emotional response to the VIPVIZA intervention has been previously published [15, 16].

Both the intervention and control groups underwent risk factor measurements and questionnaires at the 1-year follow-up. At the 1-year follow-up, the same CVD risk factors were measured as at baseline (blood pressure, lipids, fasting glucose, BMI) and with the same methodology as at baseline. Also, the identical questionnaire regarding smoking habits, physical activity, and alcohol use was answered. The results were fed back with a structured written form to participants in both the intervention—and the control groups and to their primary care physicians. This also included structured recommendations for follow-up, lifestyle modification, and, if needed according to guidelines, contact with the physician for further evaluation. The research team was not involved in preventive measures at the health care centres. The general practitioners acted according to their own judgements and existing guidelines for prevention [9]. At the 3-year follow-up, the same risk factor measurements, questionnaires, and an ultrasound examination were repeated. This time, the intervention was given to both groups and their physicians, due to ethical reasons. Further details about VIPVIZA procedures, inclusion, and exclusion criteria have previously been published [8].

## Data sources

In addition to data from VIPVIZA baseline, 3-year examination date, all dispensed CVD medications to the individuals enrolled in VIPVIZA were made available via the Swedish prescribed drug registry [17], which contains information on dispensing of all prescribed drugs from all Swedish pharmacies. This information was used to identify the individuals’ dispensing of statins, used for calculation of adherence. Included drugs were identified by ATC codes (C10AAxx, C10B, C10BA, C10BX), statins.

## Statistical analysis

All calculations of adherence were performed in R version 4.1.3 [18] using the AdhereR package [19]. Adherence was measured by continuous multiple interval measures of medication availability (CMA). Specifically, CMA6 as implemented in AdhereR version 0.7.0 was used throughout this study. The CMA measures differ with respect to (a) how the observation window is handled, (b) whether time before first and after last medication event are included, (c) if the measure is capped at 1 (100%) or not, (d) how medication oversupply is handled, discarded, or carried over into the next medication event, and finally, (e) if a medication supply can be carried into the observation window.

The adherence measure, CMA6, allows to carry over the remaining medication supply, at a new dispensing event. Dose changes were also considered so that the duration of the remaining supply was recalculated if a dose change was included in a dispensing event. At a drug change, all supplies of previous drug were discarded.

Since our observation window is large, 5 years for many participants, one summary measure for the entire period would not be valuable. Instead, a sliding window CMA6 was applied to get better resolution in the change of adherence over time. Starting 270 days before baseline examination, CMA6 was calculated for the next 180-day period, this time window then slides forward 90 days, and CMA6 was again calculated for that 180-day window. Thus, two consecutive values of adherence were based on 50% shared data. The rationale for this was to smooth the adherence measure by covering multiple prescription periods, normally 90 days in Swedish health care. This was made for all participants and an average CMA6 for each group, intervention, and control was calculated with 90 days between values. An adherence of 0.8 or above can be deemed as good adherence to statins [20].

## Results

### Overall, intervention vs control

**Fig. 2:**
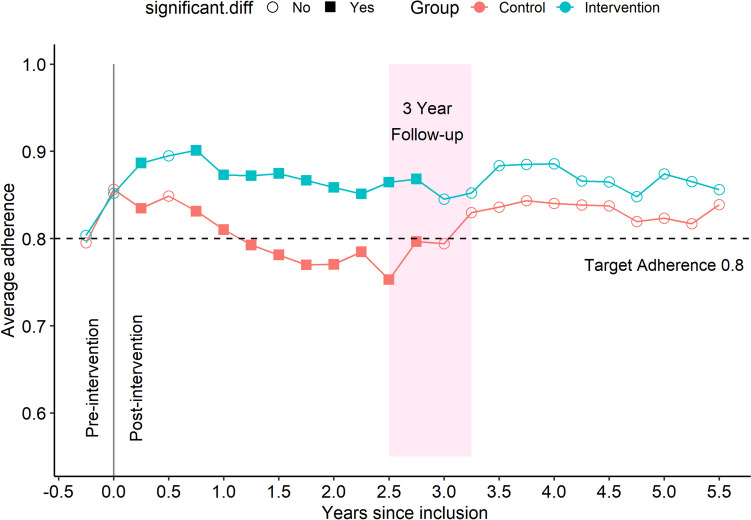
Average adherence in intervention group (blue line *n* = 263) and control group (red line *n* = 249). Adherence (CMA6) is calculated for a 180-day window and is presented as a dot at the end of the 180 days in that window. The 180 days window then slides 90 days forward and the next value is calculated. An independent samples *t*-test is calculated for the difference between intervention and control at each timepoint. Significant differences (*p* < 0.05) are indicated with filled squares, while non-significant differences are indicated with unfilled circles

Comparing the control group against intervention group, in Fig. 2, the intervention group initially reached a higher average adherence and maintained a higher adherence, while the controls declined to below 0.8. At the 3-year follow-up, which included a repeated intervention for the intervention group and a first-time intervention in the control group, an increase in adherence was observed in both groups. Both groups maintained an average adherence above 0.8 for the remainder of follow-up time. The different symbols, ring and square, in Fig. 2, indicate whether a *t*-test for the difference between control and intervention groups was significant at the 5% level. The test was performed at each timepoint and not corrected for multiple testing.

### Females, intervention vs control

**Fig. 3:**
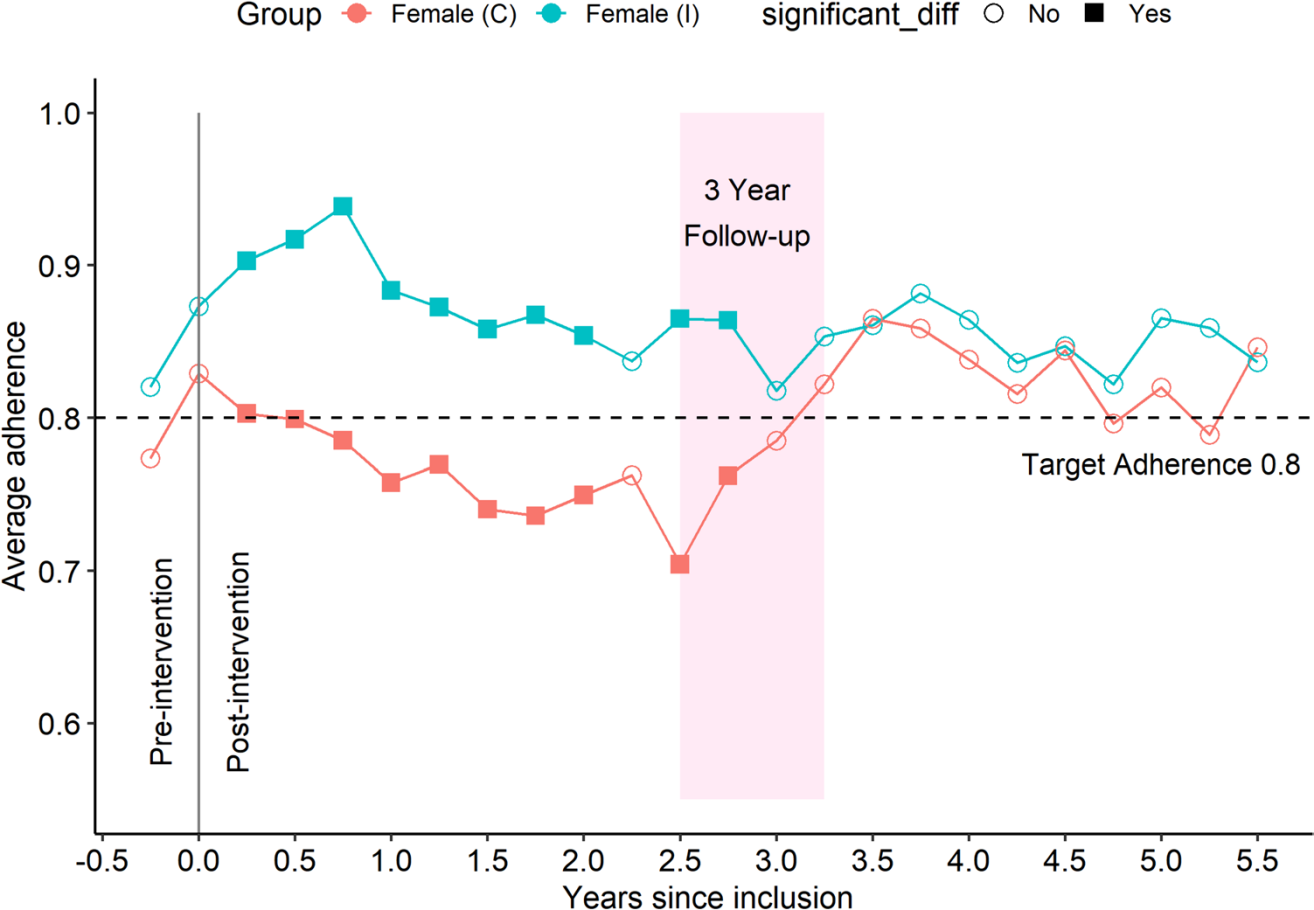
Subgroup female, intervention vs control. Average adherence in intervention group (blue line *n* = 104) and control group (red line *n* = 104). Adherence (CMA6) is calculated for a 180-day window and is presented as a dot at the end of the 180 days in that window. The 180 days window then slides 90 days forward and the next value is calculated. An independent samples *t*-test is calculated for the difference between intervention and control at each timepoint. Significant differences (*p* < 0.05) are indicated with filled squares, while non-significant differences are indicated with unfilled circles

Figure 3 illustrates women in the control and intervention group, respectively. The intervention group showed a sharp increase initially in conjunction with intervention and a slow decline towards 0.8 over 3 years. When the control group crossed over to intervention at the 3-year follow-up, they showed a similar trajectory as initially found in the intervention group. See Fig. 5 left panel for a comparison of trajectories of average adherence in the female intervention and control groups in conjunction with their respective first intervention.

### Males, intervention vs control

**Fig. 4:**
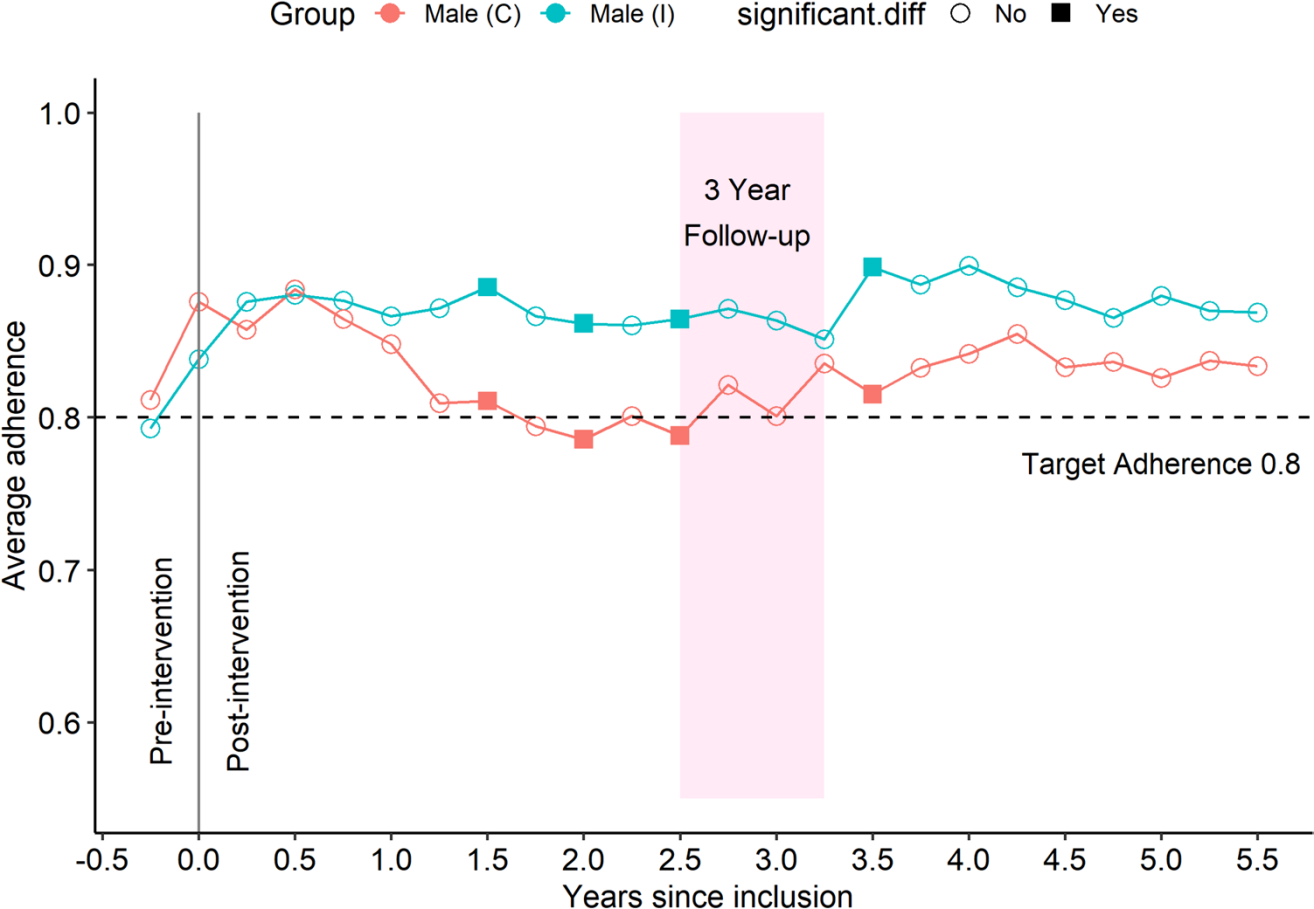
Subgroup male, intervention vs control. Average adherence in intervention group (blue line *n* = 159) and control group (red line *n* = 145). Adherence (CMA6) is calculated for a 180-day window and is presented as a dot at the end of the 180 days in that window. The 180 days window then slides 90 days forward and the next value is calculated. An independent samples *t*-test is calculated for the difference between intervention and control at each timepoint. Significant differences (*p* < 0.05) are indicated with filled squares, while non-significant differences are indicated with unfilled circles

**Fig. 5:**
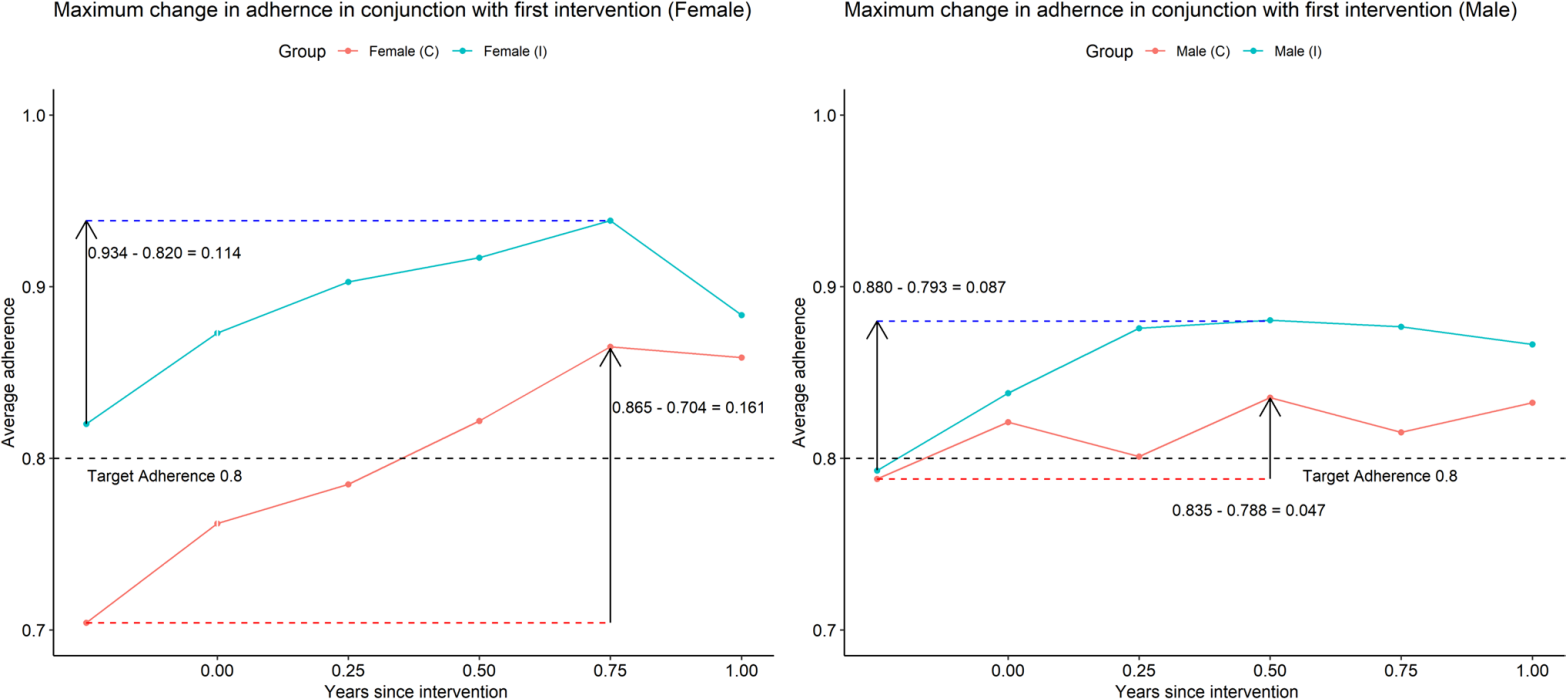
Effect from first intervention. Left panel female, right panel male. The intervention group (I) received intervention at time 0 while control group (C) got their first intervention within a timespan ranging from 2.5 to 3.25 years later. For easier comparison of the trajectory of change in adherence, these two series of observations are placed on a common time-axis, one for each sex. The largest change in average adherence over a year is marked for both series. Since only a proportion of the (C) group had their intervention at 2.5 years study time after inclusion (the first observation in the female(C) line), the first observation before intervention for (I) group is also included to show the similar trajectories for both groups in conjuncture with the intervention

Figure 4 illustrates men in subgroups for intervention and control. The male intervention group had a higher average adherence than the control group for most of the 5 years. Adherence in the intervention group was high and stable over the first 3 years but increased further after the re-intervention. The control group also showed an increase in conjuncture with the 3-year follow-up and cross-over to intervention but never reached the level in the intervention group. Figure 5, right panel, illustrates the effect of first intervention in intervention and control groups for males.

### Intervention group, male vs female

Both sexes had a high and stable adherence, but the initial intervention effect was more pronounced in the female group. See Fig. 5.

### Control group, male vs female

The control group received usual clinical management during the first period, approximately 3 years. The males demonstrated a higher degree of adherence to their prescribed statin treatment for the initial 3-year period. At the 3-year follow-up when the control group crossed over to intervention, the previously reported sharp increase in the female group completely removed this trend of a difference between sexes.

## Discussion

The intervention affected statin adherence in the total study population. It was also clear that repeating the intervention periodically helped maintaining the effect over time. Women had the strongest intervention effect of all subgroups. This might be due to perception bias that CVD is to a larger extent a problem in men, even though the lifetime CVD risk is similar across sexes [21]. At the same time, males have been shown to perceive their MI risk to be lower than females, which also partly could explain the greater increase in adherence after intervention in females shown here [22, 23]. The pictorial information about presence of plaque and vascular age (risk communication), paired with motivational talk about exercise, diet, and living habits, has a higher impact on women. The relatively smaller effect on adherence in the male participants might be due to the general perception that CVD risk is more serious for men and that is why the male participants exhibit a higher adherence already at baseline. Several earlier studies have highlighted the sex difference to reduce disparities in risk awareness, clinical care, and adherence to CVD risk reducing treatment or lifestyle changes [24, 25]. In a meta-analysis, it was observed that women were less likely to get cardiovascular medication prescribed in primary care [26]. Even regarding secondary prevention following a major cardiovascular event, the use of preventive medication is lower for women [27].

The key factors affecting adherence were identified previously by Martin et al. [28]: “patients who participate in discussions of behavioural strategies with their doctor are more likely to adhere”, “Patients who are informed and affectively motivated are also more likely to adhere to their treatment recommendations”, “Patients who feel that their physicians communicate well with them and actively encourage them to be involved in their own care tend to be more motivated to adhere”.

The intervention in VIPVIZA targeted all the above-mentioned factors as previously described [9, 12, 15]. The intervention also targeted the physicians, who received pictorial information about the patient’s plaque, vascular age, and other risk factors such as cholesterol and blood pressure so that Framingham risk score or SCORE could be used to estimate future cardiovascular risk. Attached to the patient-related information to the physicians, guidelines for preventive treatment of patients with silent arthrosclerosis were also included. We have previously shown a clear increased rate of prescriptions of statins after the VIPIVZA intervention [11, 29].

The intervention to the participants consisted of pictorial risk information sent by mail, followed up by a phone call from a research nurse. The phone call played an important role to address concerns and anxiety about the risk information as well as to motivate to preventive measures. Within VIP, all participants, both intervention and control group, had a motivational interview focusing on their risk factors and how these can be addressed via medication and lifestyle changes. The individual risk assessment with pictorial information, in the intervention group, was performed to aid/enhance the risk communication, by making the information more concrete and patient-centred, and to motivate the patient by clearly stating that this is a dynamic process that can be delayed or even reversed. A visualisation of your arteries gives a higher risk perception than any number on an, for most patient’s, abstract risk score. How this was experienced by and affected the participants was previously described [12, 15, 16]. How the intervention affects physicians’ prescriptions of statins and facilitates patient interaction and shortens time to initiation of statins has earlier been described [9, 11]. In recent studies, the difference between men and women regarding both the patients’ and physicians’ view of CVD risk was highlighted. These differences contribute to women having lower chance to be recommended statin treatment and a higher risk of being non-adherent [4, 7].

The present intervention is multifaceted, with a personalised pictorial information intervention addressing both the participant and the treating physician, raising awareness of the risk. This is combined with motivational talk with a nurse explaining potential loss or gain from implementing changes and strategies to cope with the changes. Therefore, it is impossible to distinguish any singular component of the programme as responsible for the large, sustained increase in adherence to statins, particularly for women. The programme as a package successfully addresses the often suboptimal CVD risk management in primary care.

## Data Availability

The data underlying this article were provided by VIPVIZA by permission. Data will be shared on reasonable request to VIPVIZA. Project web: https://www.umu.se/en/research/projects/visualization-of-asymptomatic-atherosclerotic-disease-for-optimum-cardiovascular-prevention.-a-population-based-rct-within-the-vip--vipviza-/.

## Declarations

### Disclaimer

The funders of the study had no role in the study design, data collection, data interpretation, or writing of the report. The corresponding author had full access to all the data in the study and had final responsibility for the decisions to submit for publication.
